# Assessing craving in cocaine and cocaine base paste users: validation of the Cocaine Craving Questionnaire-Brief in a Chilean sample

**DOI:** 10.1186/s13722-026-00647-5

**Published:** 2026-01-22

**Authors:** Felipe Bustamante, Soledad Labbé, Tomás Arriaza, Ivelisse Huerta, Miguel Cordero Vega, María Elena Alvarado, Alvaro Vergés

**Affiliations:** 1 Universidad de los Andes, Escuela de Psicología, Santiago, Chile; 2 Universidad de Chile, Escuela de Salud Pública, Santiago, Chile; 3 Universidad del Desarrollo, Santiago, Chile; 4 University of Bristol, Bristol, England; 5 Núcleo Milenio para Mejorar la Salud Mental de Adolescentes y Jóvenes, Santiago, Imhay Chile

**Keywords:** CCQ-Brief, Cocaine, Cocaine base paste, Craving, Psychometric properties

## Abstract

**Background:**

One of the principal components of substance use treatment is the assessment of craving, as it is highly associated with relapse after treatment. The Cocaine Craving Questionnaire-Brief (CCQ-Brief) has emerged as a commonly used tool in the field, relevant in a context like South America, where cocaine and cocaine base paste (CBP) consumption are critical public health issues.

**Objective:**

This study aimed to validate the CCQ-Brief in a Chilean sample, exploring for the first time its applicability in both cocaine and CBP use.

**Methods:**

Adults in substance use treatment (*N* = 439, 71,3% male) completed the CCQ-Brief, assessing craving for cocaine and CBP over the past 30 days. Confirmatory factor analysis (CFA) was conducted to test the CCQ-Brief one-factor structure. Convergent validity was analyzed through correlations with related constructs, including impaired control, impulsivity facets, frequency of cocaine/CBP use and related symptomatology.

**Results:**

CFA supported CCQ-Brief one-factor structure, with high internal consistency (α = 0.92 and ω = 0.94 for the 10-item version). Factorial invariance analysis showed that the CCQ-Brief performs equivalently across cocaine and CBP users. Craving scores were moderately to strongly correlated with impaired control dimensions, impulsivity facets, cocaine/CBP use and related symptomatology.

**Conclusions:**

These results show evidence of the CCQ-Brief as a reliable and valid instrument for general craving of cocaine and CBP in a Chilean context, being the first version of the CCQ-Brief that confirms its unifactorial dimension with all its 10 items in the country.

**Supplementary Information:**

The online version contains supplementary material available at 10.1186/s13722-026-00647-5.

Cocaine ranks among the top three most widely used illicit drugs globally, following cannabis and amphetamines, with an estimated 22 million individuals using it at least once in 2023 [34]. South America, the world’s largest producer of cocaine, has seen an increase in both the confiscation and consumption of cocaine and its derivative, cocaine base paste (CBP), which remains particularly concentrated in the region [4, 34]. In Chile, the prevalence of cocaine and CBP use in the last year among individuals aged 12 to 65 is 0.9% and 0.3%, respectively [29]. Moreover, lifetime cocaine use has increased compared to previous years [20, 29]. Notably, among those treated in outpatient and residential drug programs, nearly half sought treatment for cocaine-related stimulants (cocaine: 19.2%; CBP: 28.6%) [28]. These trends involve significant public health challenges, requiring comprehensive strategies in both clinical and social contexts.

CBP and cocaine represent different stages of cocaine hydrochloride production. CBP has a higher addictive potential and stronger effects than pure cocaine, largely due to its composition, combustion byproducts, and the route of administration (e.g., smoking;[35]). Despite their differences, both substances pose severe health and social risks, including increased cardiovascular disease, susceptibility to infections, and societal challenges such as crime and reduced social cohesion [8, 33]. Notably, CBP should not be confused with crack, a drug often consumed in Europe and North America, as it results from different processes of adulterating cocaine hydrochloride.

One of the principal components of substance use disorders treatment is the assessment and clinical management of craving, defined as an intense desire or urge to use a substance [7]. Craving plays a critical role in the onset and persistence of addiction [7, 15] and often serves as a barrier to sustained abstinence and successful rehabilitation, as it has a well-known association with relapse [15, 30]. Measuring craving is therefore essential for developing effective interventions and treatment strategies. Moreover, research has shown that craving is associated with other relevant clinical variables, such as impulsivity [12] and impaired control [26].

The Cocaine Craving Questionnaire-Brief (CCQ-Brief) ([31] adapted from [32]) is a widely used instrument designed to assess general craving in cocaine users. The CCQ-Brief was derived from the original 45-item Cocaine Craving Questionnaire-Now (CCQ-Now; [32]) to address practical limitations associated with the length of the full measure. The CCQ-Now, a psychometrically robust instrument, assesses cocaine craving through a multifaceted approach. Factor analysis of the CCQ-Now revealed four factors accounting for 80% of the variance in the observed data, with Factor 1 (general craving) alone explaining 54% of the variance. To create the CCQ-Brief, the 10 items that loaded most heavily on this primary factor were selected, creating a one-factor scale that showed a strong internal consistency (Cronbach’s Alpha of 0.90). The instrument can be completed in under two minutes, features straightforward items, and allows for quick scoring and interpretation [31].

Despite its widespread use [11, 14, 17, 19, 37], validation studies of the CCQ-Brief remain limited, especially in diverse cultural and linguistic contexts. Most validations have focused on internal consistency and correlations with other instruments, with generally good results and evidence of its convergent validity [3, 13, 21, 23, 31]. However, only [3] have examined its factorial structure using confirmatory factor analysis (CFA). In their study, the initial model showed poor fit (RMSEA = 0.18 [90% CI: 0.15–0.21]), and the authors subsequently removed two reverse-scored items based on their item–total correlations (item 4 = 0.33; item 7 = 0.58), resulting in a one-factor, 8-item version with improved fit (RMSEA = 0.08 [90% CI: 0.01–0.12], CFI = 0.99, TLI = 0.99). While these items were considered by the authors to have comparatively weak item–total correlations, they assess theoretically relevant aspects of craving. Moreover, the relatively small sample size of that study (*n* = 102), together with the continued use of the 10-item CCQ-Brief in subsequent studies across different cultural settings [17, 19], underscores the need for further research examining the full scale.

The CCQ-Brief has been typically utilized in research as a mean score [11, 14, 16, 17, 19, 25, 37], implicitly assuming a unidimensional construct. Apart from the Colombian validation study by [3], no other validation of the CCQ-Brief has been conducted in the South American context. Moreover, to our knowledge, there are no existing instruments specifically designed to assess CBP craving. Given the similarities in the neurobiological and psychological mechanisms underlying craving across cocaine and CBP [5, 18], and the general nature of the CCQ-Brief items -which may effectively capture the global construct of craving independent of the specific drug referenced [31]-, the adaptation and validation of the CCQ-Brief for the assessment of CBP craving presents a valuable opportunity to address this gap. This study aims to evaluate the reliability and validity of the Spanish adaptation of the CCQ-Brief for both cocaine and CBP use in a Chilean sample, providing a critical tool for tackling a pressing public health issue.

## Methods

### Participants

This study used data from two studies on substance use disorder treatment conducted by research teams led by the corresponding author (A.V.). One study aimed to validate the Impulsive Behavior Scale (UPPS-P) and other instruments [2], whereas the other aimed to explore the effect of impulsivity on substance use outcomes among patients in treatment. Participants were recruited from outpatient and residential substance use treatment facilities in Santiago, Chile. Adult participants who completed the CCQ-Brief were selected from each study, resulting in a final analytic sample.

Participants with psychiatric disorders or substance-induced psychiatric symptoms that were not under control (e.g., acute psychotic symptoms, active suicidal risk) were excluded from the study. This assessment was conducted by the clinical staff at each treatment center before recruitment to ensure that only clinically stable patients were invited to participate. The filtering process took place prior to data collection, and as such, the research team did not directly assess or record the number of individuals excluded on this basis.

Questionnaires were administered in a self-report format, where participants chose between self-administration or administration by a trained research assistant. All participants provided informed consent prior to study activities.

All procedures for this study received approval from the Institutional Review Board of Universidad Católica and the Institute of Nutrition and Food Technology (ID: 150701005).

### Measures

CCQ-Brief: The CCQ-Brief was translated into Spanish and then back-translated by a native English speaker. The current adaptation assessed cocaine and CBP craving during the last 30 days. To allow assessment of CBP use, the word “cocaine” was replaced with “cocaine/cocaine base paste” in each question. The items are scored on a four-point Likert-type scale from “Never” (1) to “Always” (4), with 8 items positively keyed and two reverse-keyed items (items 4 and 7). For exploratory testing, two additional items were included, considering the Chilean context: item 11 (“*I feel a strong desire to consume cocaine/CBP”)* and item 12 (“*I have such a strong desire to consume cocaine/CBP that I can’t think of anything else*”). Items 11 and 12 were added as they are frequently used in epidemiological surveillance measuring cocaine and CBP use in Chile by the National Service for the Prevention and Rehabilitation of Drug and Alcohol Use [29].

Impaired Control Scale (ICS): The ICS is a 25-item widely used questionnaire to assess impaired control over alcohol and other drugs. It is composed of 3 factors that measure different aspects of a person’s ability to control alcohol or drug use: Attempted Control, Failed Control, and Perceived Control. The first two factors use a Likert-type scale, ranging from “Never” (1) to “Always” (4). Perceived Control, the third factor, has a response range from “Strongly Disagree” (1) to “Strongly Agree” (4). Cronbach’s alpha coefficients of the three factors showed good internal consistency (from 0.84 to 0.95) in the original version of the scale [10].

Impulsive Behavior Scale (UPPS-P): The UPPS-P is a 59-item Likert-type questionnaire developed to address impulsivity facets. The original scale is composed of 5 factors: Negative Urgency, Lack of Planning, Lack of Perseverance, Sensation Seeking, and Positive Urgency. For this study, we considered a 3-factor structure that combines Negative Urgency and Positive Urgency into one factor, and Lack of Planning and Lack of Perseverance into a second factor. This structure has been validated in a Chilean clinical sample, presenting good internal consistency (Omega categorical: 0.84 to 0.93) [2].

Cocaine/CBP use symptomatology: A 11-item “Yes/No” questionnaire developed by SENDA (2022) was used. This questionnaire includes questions based on international classifications, such as the DSM-5 and CIE-10. A summative score ranging from 0 to 11 was created, with higher values representing a stronger symptomatology.

Frequency of cocaine/CBP use: Cocaine and CBP use frequency was assessed with a single item for each substance: “Thinking only about the last 30 days, how many days during the month have you used cocaine?” and “Thinking only about the last 30 days, how many days during the month have you used CBP?”

### Data analysis

We performed a descriptive analysis of the sample considering sociodemographic and clinical variables.

To assess the validity of the Cocaine Craving Questionnaire Brief (CCQ-Brief), we conducted a one-factor Confirmatory Factor Analysis (CFA) of the 10 and 12-item versions, using Mplus 8 [22]. All items were treated as ordinal; therefore, the Weighted Least Square Mean and Variance Adjusted Estimator (WLMSV) was used. Given that participants were enrolled in different treatment programs, these programs were included as a cluster variable in the analysis to account for nesting effects. Additionally, a model including a method factor was tested to assess the presence of reverse-scored items.

After conducting CFA, metric, configural, and scalar measurement invariance analyses were carried out across type of substance use (cocaine vs. CBP) using the model with best fit indices (Model 3). Invariance between measurements was determined when ΔCFI < 0.01 [6]. We excluded participants who consumed both cocaine and CBP for invariance testing to avoid group contamination; these individuals accounted for 4.55% (20) of the total sample.

To determine the internal consistency of the 10 and 12-item Spanish CCQ-Brief, Cronbach’s α was calculated for the whole scale. Green & Yang’s (2009) categorical omega was also used, considering the categorical nature of the items.

We also tested the convergent validity of the selected CCQ-Brief models using Pearson’s correlation between the CCQ-Brief, the three dimensions of the ICS, the three factors of the UPPS-P scale, and substance use frequency and symptomatology. Analyses were conducted separately based on the main substance of use of the participants (cocaine and CBP). The correlations considered the CCQ-Brief as a latent factor, whereas the other variables were scored as the mean of each factor.

## Results

### Demographic and clinical characteristics of the sample

This study used data from two samples (see Methods section for details). Sample 1 included 316 patients (adults and adolescents) recruited from nine outpatient and residential substance use treatment facilities in Santiago, Chile. A subsample of adult participants who completed the CCQ-Brief was selected, resulting in 173 individuals aged 18–60 years (mean = 35.59; SD = 9.34), with 69.9% (*n* = 121) male.

Sample 2 comprised 327 adult patients recruited from 12 outpatient and residential treatment facilities in Santiago, Chile. A subsample of 266 participants who completed the CCQ-Brief was included, aged 19–59 years (mean = 34.29; SD = 9.09), with 72.2% (*n* = 192) male. The final study sample was comprised of 439 patients (mean age = 34.80; SD = 9.20; 71.3% male).

The demographic characteristics of the total sample are summarized in Table 1. CBP and cocaine were the sole primary substances in 53.5% and 35.5% of the cases, respectively. The remaining participants were classified into mixed-substance categories, including treatment for cocaine with other substances such as alcohol, marijuana, and/or pills (3.19%); CBP with other substances (3.19%); both cocaine and CBP (3.64%); or combined cocaine, CBP, and other substances (0.91%). The mean score of the CCQ-Brief was 1.86 (SD: 0.79) and 1.84 (SD: 0.80) for the 10 and 12-item versions, respectively. While no validated clinical cut-off exists to interpret the CCQ-Brief score, the scale considers scores from 1 to 4, with higher scores suggesting a higher level of craving. Our results are in the lower half of the possible range, indicating relatively low-to-moderate craving intensity in our sample.

**Table 1:** Demographic and clinical characteristics of the sample

Variable	Values
Age (mean) (sd)	34.80 (9.20)
Gender % (n)	
Male	71.3% (313)
Female	28.5% (125)
Missing	0.22% (1)
Education % (n)	
C/I Primary education	17.08% (75)
C/I Secondary education	53.1% (233)
C/I Higher Professional Technical Education	16.85% (74)
C/I College degree	12.75% (56)
Missing	0.22% (1)
Substance for treatment % (n)	
Cocaine only	35.5% (156)
Cocaine (main) and other substance	3.19% (14)
CBP only	53.5% (235)
CBP (main) with other substance	3.19% (14)
Cocaine and CBP	3.64% (16)
Cocaine (main), CBP and other Substance	0.91% (4)
CCQ-Brief (mean) (sd)	
10 items	1.86 (0.79)
12 items	1.84 (0.80)
UPPS-P (mean) (sd)	
Conscious	2.22 (0.43)
Urgency	2.75 (0.48)
Sense seeking	2.68 (0.54)
ICS (mean) (sd)	
Attempted Control	2.69 (0.93)
Failed Control	3.02 (0.62)
Perceived Control	2.41 (0.66)
Number of days using each substance in the past 30 days (mean) (sd)	
Cocaine	9.88 (10.17)
CBP	15.33 (10.62)

Note: sd = standard deviation; n= number of observations; CBP = Cocaine Base Paste; CCQ-Brief= Cocaine Craving Questionnaire-Brief; ICS= Impaired Control Scale; UPPS-P= Impulsive Behavior Scale; C/I= complete or incomplete.

Additionally, among individuals who reported past-year use, cocaine was used on an average of 9.88 days (SD: 10.17) in the past month, while CBP was used on an average of 15.33 days (SD: 10.62).

Regarding the UPPS-P, participants presented a mean score of 2.75 for the urgency dimension, 2.68 for the sensation seeking dimension, and 2.22 for the Conscientiousness dimension. Finally, for the dimensions of the Impaired Control Scale (ICS), the mean score of failed control was 3.02 (SD: 0.62), for attempted control it was 2.69 (SD: 0.93), and 2.41 (SD: 0.66) for perceived control. The mean (SD) scores for the instruments are shown in Table 1.

### Model comparison

Model comparison based on fit indices is shown in Table 2. Initially, we ed both theoretical models with all intended factors. However, one of the model fit indices, the Root Mean Square Error of Approximation (RMSEA), indicated a suboptimal fit, suggesting the need for model refinement. As such, a subsequent CFA was performed, incorporating a single modification index based on the highest modification suggestion considering the correlation between items 1 and 2 for each model. Both items present similar content (“I want cocaine/CBP so bad I can almost taste it” and “I have an urge for cocaine/CBP”) and were part of the same factor in the original version of the CCQ [32]. This adjustment was made to improve model fit while maintaining the theoretical integrity of the scale. The decision to include only one modification index was guided by the principle of making the model more parsimonious without overfitting the data to sample-specific characteristics. The final refined models showed acceptable fit indices and were used for subsequent analyses.

**Table 2:** Model fit indices for the 10 and 12-items Spanish-adaptation of the CCQ-Brief

Model	*N*° of ítems	χ² (df)	RMSEA (90% CI)	CFI	TLI	SRMR
1	10	239.532 (35)	0.116 (0.103; 0.131)	0.980	0.975	0.053
2	12	317.978 (54)	0.106 (0.095; 0.118)	0.987	0.984	0.047
3	10 (MI)	142.062 (34)	0.086 (0.072; 0.101)	0.990	0.986	0.042
4	12 (MI)	234.998 (53)	0.089 (0.078: 0.101)	0.981	0.989	0.040
5	10 (Method)	236.775 (34)	0.118 (0.104; 0.132)	0.981	0.974	0.049
6	12 (Method)	317.537 (53)	0.108 (0.096; 0.119)	0.987	0.984	0.044

Note:MI= modification index between items 1 and 2; Method= Method factor considering reverse items 4 and 7; CCQ-Brief= Cocaine Craving Questionnaire-Brief; χ²= chi-square; df= degrees of freedom; RMSEA= Root Mean Square Error of Approximation; CFI= Comparative Fit Index; TLI= Tucker–Lewis Index; SRMR= Standardized Root Mean Square Residual

As shown in Table 2, after including the residual correlation, models 3 and 4 exhibited the best fit, with acceptable goodness-of-fit indices (model 3: RMSEA = 0.086 [90% CI: 0.072–0.101], CFI = 0.99, TLI = 0.98; model 4: RMSEA = 0.089 [90% CI: 0.078–0.101], CFI = 0.98, TLI = 0.98). Among all fit indices, only RMSEA exceeded the conventional threshold of 0.08; however, values between 0.08 and 0.10 are considered “mediocre but acceptable” [1, 27], whereas the CFI and TLI values indicate excellent fit. All models also showed SRMR values below 0.06, and all factor loadings were significant and above 0.60, ranging from 0.66 to 0.94 (see Table 3; detailed factor loadings for all tested models are reported in Table S6). Based on these criteria, models 3 and 4 were selected for subsequent analyses.

**Table 3:** Factor loadings for the 1-factor structure of the 10 and 12-items CCQ-Brief Spanish-adaptation

In the last 30 days, I have felt or thought:	Model 3	Model 4
1. I want cocaine/CBP so bad I can almost taste it.	0.662	0.684
2. I have an urge for cocaine/CBP.	0.868	0.875
3. I am going to use cocaine/CBP as soon as possible.	0.910	0.879
4. I think that I could resist using coke/CBP (reverse).	0.672	0.665
5. I crave cocaine/CBP.	0.941	0.940
6. All I want to use is cocaine/CBP.	0.937	0.943
7. I have no desire for cocaine/CBP. (reverse)	0.716	0.714
8. Using cocaine/CBP would make things seem just perfect.	0.763	0.764
9. I will use cocaine/CBP as soon as I get the chance.	0.931	0.919
10. Nothing would be better than using cocaine/CBP.	0.912	0.911
11. I feel a strong desire to use cocaine/CBP	–	0.932
12. I have such a strong desire to consume cocaine/CBP that I can’t think of anything else	–	0.939

Note: All loadings were significant under *p* < 0.01; CBP = Cocaine Base Paste; CCQ-Brief = Cocaine craving questionnaire-Brief

### Measurement invariance and latent factor means differences across substances

A multi-group confirmatory factor analysis was conducted to test for measurement invariance of models 3 and 4 across two groups based on the main drug consumed (cocaine vs. CBP). Results indicated that the configural, metric, and scalar models presented an adequate fit, with a slightly better fit for RMSEA, and a more than acceptable fit on TLI, CFI, and SRMR (see Table 4).

**Table 4:** *M*odel fit for invariance for the 10 and 12-items Spanish-adaptation of the CCQ-Brief

Model	*N*° of ítems	χ² (df)	RMSEA (90% CI)	CFI	TLI	SRMR
Configural	10	153.381 (68)	0.081 (0.064; 0.098)	0.993	0.991	0.048
Metric	10	163.443 (77)	0.076 (0.060; 0.093)	0.993	0.992	0.050
Scalar	10	183.291 (96)	0.069 (0.053; 0.084)	0.993	0.993	0.051
Configural	12	243.570 (106)	0.082 (0.069; 0.096)	0.994	0.992	0.046
Metric	12	255.206 (117)	0.078 (0.065; 0.091)	0.994	0.993	0.047
Scalar	12	275.407 (140)	0.071 (0.058; 0.083)	0.994	0.994	0.044

Note: χ²= chi-square; df= degrees of freedom; RMSEA= Root Mean Square Error of Approximation; CFI= Comparative Fit Index; TLI= Tucker–Lewis Index; SRMR= Standardized Root Mean Square Residual

The ΔCFI values across all model comparisons were below the recommended threshold of 0.01, confirming scalar invariance across cocaine and CBP consumption groups. Specifically, comparisons between the metric and configural models (Model 3: Δχ² = 12.041, df = 9, *p* = 0.211, ΔCFI = 0; Model 4: Δχ² = 15.334, df = 11, *p* = 0.1677, ΔCFI = 0) and between the scalar and metric models (Model 3: Δχ² = 34.087, df = 19, *p* = 0.0136, ΔCFI = 0; Model 4: Δχ² = 40.978, df = 23, *p* = 0.0119, ΔCFI = 0) showed no significant change in model fit, supporting invariance at both levels.

The estimated latent mean difference was considered as small (Δ = 0.213, *p* = 0.057). Its confidence interval included zero, suggesting limited evidence for a meaningful difference in craving between groups.

### Internal consistency

Cronbach’s α for both the 10 and 12-item CCQ-Brief showed high internal consistency (0.92 and 0.94). Additionally, all items correlated highly with the total score (ranging from 0.64 to 0.89 and 0.67 to 0.89, respectively). Green & Yang’s [9] categorical ω was 0.94 for the 10-item version, and 0.96 for the 12-item version.

### Convergent validity

Table 5 illustrates the correlation between different constructs and the CCQ-Brief (Model 3) for each main substance. For cocaine users, the CCQ-Brief showed strong correlations with several impulsivity facets and control dimensions. Among the UPPS-P factors, craving was strongly associated with urgency (*r* = 0.40, *p* < 0.01). A moderate and significant association was observed with conscientiousness (*r* = 0.25, *p* < 0.01), and sensation seeking did not show a significant association with the CCQ-Brief. On the other hand, the CCQ-Brief correlated significantly with all factors of the ICS, showing moderate to strong associations across factors. The highest correlation was with the Perceived Control (*r* = 0.55, *p* < 0.01), followed by Failed Control (*r* = 0.32, *p* < 0.01) and Attempted Control (*r* = − 0.28, *p* < 0.01). Finally, the CCQ-Brief also presented a strong and positive correlation with frequency of cocaine use (*r* = 0.44, *p* < 0.01) and cocaine use symptomatology (*r* = 0.35, *p* < 0.01) in this group.

**Table 5:** Correlations between variables and CCQ-Brief (Latent) Spanish-adaptation

Cocaine/Cocaine base paste	1	2	3	4	5	6	7	8	9
1. CCQ-Brief (M3)	-	-0.38	0.34	0.54	0.19	0.32	0.26	0.41	0.34
2. Attempted Control	-0.28	-	-0.27	-0.33	-0.15	-0.05	-0.11	-0.36	-0.15
3. Failed Control	0.32	-0.23	-	0.53	0.17	0.23	0.16	0.22	0.51
4. Perceived Control	0.55	-0.22	0.39	-	0.25	0.23	0.13	0.31	0.24
5. Consciousness	0.25	-0.29	0.06*	0.21	-	0.38	-0.05	0.07	0.07
6. Urgency	0.40	-0.24	0.29	0.34	0.24	-	0.32	0.15	0.026
7. Sensation seeking	0.07*	-0.16	0.15	0.01*	-0.10*	0.40	-	0.17	0.019
8. Cocaine/CBP frequency use	0.44	-0.26	0.34	0.35	0.25	0.17*	0.02*	-	0.21
9. Cocaine/CBP symptoms	0.35	-0.19	0.60	0.39	0.05*	0.24	0.21	0.26*	-

*Note 1*: Correlations above the diagonal correspond to the group cocaine base paste (CBP) users, while correlations below the diagonal correspond to the group cocaine users.*Note 2*: * not significant correlation under *p* < 0.05.

For participants who reported CBP as a main substance, correlations in the CCQ-Brief behaved similarly to their cocaine users counterparts: The CCQ-Brief showed a strong correlation with urgency (*r* = 0.32, *p* < 0.01), and a moderate correlation with consciousness (*r* = 0.19, *p* < 0.01), though, compared to cocaine users, the correlation with sensation seeking (*r* = 0.26, *p* < 0.01) was significant in this group. Similar correlations in magnitude and direction were also found for the ICS factors comparable to those observed in cocaine users (Perceived Control, *r* = 0.54, *p* < 0.01; Failed Control, *r* = 0.34, *p* < 0.01; and Attempted Control, *r* = − 0.38, *p* < 0.01). Finally, the same can also be said for the correlations with frequency of CBP use (*r* = 0.41, *p* < 0.01) and CBP symptomatology (*r* = 0.34, *p* < 0.01).

## Discussion

Cocaine and CBP consumption pose significant public health challenges for governments and healthcare systems, particularly in South American countries. Addressing this issue requires effective tools to measure specific aspects of substance use disorders, such as craving, a predictor of treatment outcomes [15, 30]. Our study examined the construct validity of the CCQ-Brief [31] to empirically determine if it is a reliable and valid tool for measuring craving in Chilean adults in substance use treatment. The internal consistency of the CCQ-Brief for cocaine and CBP use was high. This is in line with the few previous validations of the scale [13, 21, 23, 31]. Furthermore, categorical omega estimates [9] indicated excellent reliability for both the 10-item and 12-item versions ed in the current study. Model selection should not rely solely on global fit indices, but also on the strength and significance of factor loadings, the internal consistency of the scales, and the theoretical coherence of the model [1, 27]. As such, confirmatory factor analysis (CFA) showed a reasonable fit, validating the unifactorial structure of the CCQ-Brief across both 10 and 12-item models, with fit indices consistently within acceptable ranges, except for RMSEA, particularly when including a correlation between residuals.

These results align with the nature of the CCQ-Brief, a questionnaire derived mainly from one factor of the CCQ-Now. A method factor did not improve model fit, compared to the models that included a residual correlation. This lack of improvement may suggest that the observed data is not significantly influenced by response biases linked to item phrasing, such as positively or negatively worded items [24].

Regarding validity criteria, in both groups there was a positive and high correlation between the CCQ-Brief and the Perceived Control factor of the ICS questionnaire. Although no other study has analyzed this relationship between cocaine craving and perceived control, we expected that Cocaine and CBP craving may present a similar behavior to alcohol craving when correlated with the perceived control dimension of the ICS questionnaire. As Sanchez et al. (2020) showed, individuals with lower perceived control tend to report higher alcohol craving scores, reflecting a progression in the severity of alcohol use disorder. This relationship emphasizes how diminished perceived control contributes to increased craving, which in turn may exacerbate alcohol consumption and dependency. On the other hand, the positive correlation between urgency (UPPS-P) and craving indicates that individuals with higher emotion-driven impulsivity tend to experience stronger cocaine and cocaine base paste craving. Waddell et al. [36] found that both positive and negative urgency were directly related to heightened alcohol craving. Finally, convergent validity was confirmed, as higher levels of craving were associated with more frequent use of cocaine and CBP, as well as increased related symptomatology.

Measurement invariance of the CCQ-Brief was demonstrated at the configural, metric, and scalar levels across groups (cocaine vs. CBP). Based on these results, the scale showed consistency across substances, supporting its use for group comparisons. Our findings indicate that the CCQ-Brief is applicable to both groups in the Chilean context.

Our study presents several strengths. First, the CCQ-Brief has been validated in different cultural contexts, with most validations proving the instrument to be a useful tool capable of adapting to different languages and territories. Given that nearly half of those treated in Chilean drug programs in 2021 sought help for cocaine-related stimulants [28], this study provides crucial evidence for its applicability in the Chilean context. Second, most of the previous validations used techniques focused only on the CCQ-Brief internal validity, measuring Cronbach’s alpha and analyzing its correlation to other instruments and variables [13, 21, 23, 31]. Our study contributes by including the analysis of the CCQ-Brief factor structure through CFA, and estimating the impact of inverse items through a method factor. Third, while adapting the CCQ-Brief to the Chilean context, we also included two additional questions often used to measure craving in Chile’s clinical surveys. Our results showed that their addition yielded only marginal improvements in model fit, suggesting that the 10-item version may be sufficient for accurately assessing craving in the country. Fourth, our research makes a novel contribution by analyzing the CCQ-Brief adaptability to CBP consumers. Measurement invariance proved that the CCQ-Brief may be used to measure and compare craving between cocaine and CBP consumers in the Chilean context, providing evidence for the use of the CCQ-Brief to measure craving for CBP, a substance for which there are, to our knowledge, no available instruments for craving assessment. This further supports Sussner et al.’s [31] suggestion of adapting the CCQ-Brief for other substances of use, given the general nature of the craving construct assessed by the scale. Our work offers academia and clinical practitioners a valuable and concise tool for assessing CBP craving.

Despite its contributions, our study has limitations. Though all participants were part of a treatment program, cocaine, and CBP consumption were self-reported, with no formal clinical diagnosis. Additionally, due to the cross-sectional nature of our study, there was no test-retest application of the CCQ-Brief.

## Conclusions

To summarize, our results showed that the CCQ-Brief adaptation is a reliable and valid instrument to assess cocaine and CBP craving in the Chilean context. The CCQ-Brief provides an accurate and concise instrument, making it ideal for quick administration in clinical settings, where the assessment of craving is crucial for process and outcome monitoring.

## Appendix

CCQ-Brief (in spanish) used during interview:


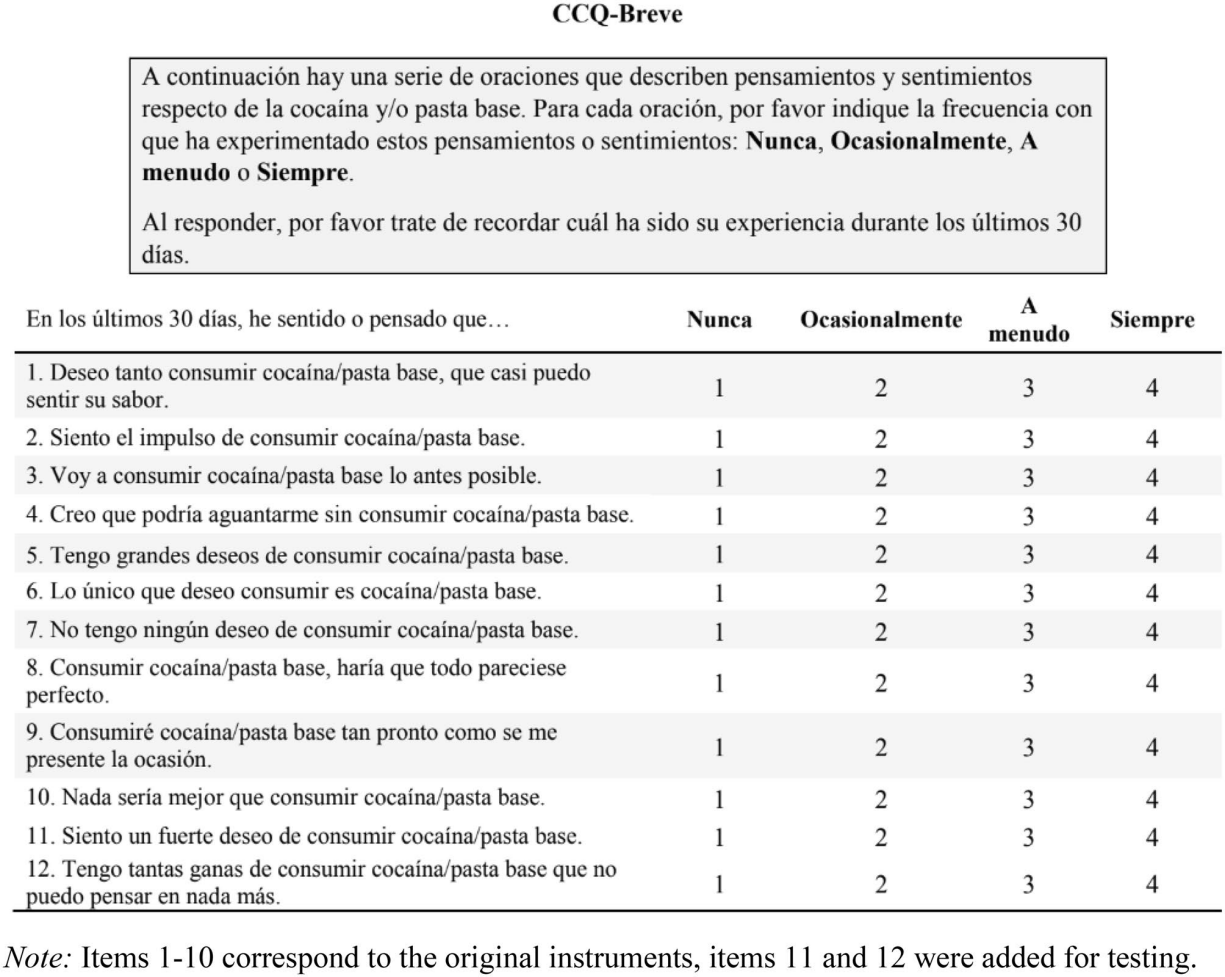


## Supplementary Information

Below is the link to the electronic supplementary material.

Supplementary Material 1

Supplementary Material 2

## Data Availability

All data generated or analyzed during this study is included in this published article and its supplementary material.
