# Supplementary material for: Assessing craving in cocaine and cocaine base paste users: validation of the Cocaine Craving Questionnaire-Brief in a Chilean sample
Source: Addict Sci Clin Pract. 2026 Jan 22;21:20. doi: 10.1186/s13722-026-00647-5 (PMC12853577; doi:10.1186/s13722-026-00647-5)
Supplement: Supplementary file 1 — Supplementary Material 1 [file 13722_2026_647_MOESM1_ESM.docx]

**Supplementary Materials**

**Table S6**

*Factor loadings for the 1-factor structure of the 10 and 12-items CCQ-Brief Spanish-adaptation*

| Question | Model 1 | Model 2 | Model 3 | Model 4 | Model 5 | Model 6 |
| --- | --- | --- | --- | --- | --- | --- |
| 1 | 0.746 | 0.749 | 0.662 | 0.684 | 0.747 | 0.750 |
| 2 | 0.918 | 0.905 | 0.868 | 0.875 | 0.919 | 0.906 |
| 3 | 0.906 | 0.877 | 0.910 | 0.879 | 0.907 | 0.878 |
| 4 (reverse) | 0.669 | 0.663 | 0.672 | 0.665 | 0.658 | 0.655 |
| 5 | 0.931 | 0.937 | 0.941 | 0.940 | 0.931 | 0.938 |
| 6 | 0.928 | 0.939 | 0.937 | 0.943 | 0.929 | 0.939 |
| 7 (reverse) | 0.707 | 0.708 | 0.716 | 0.714 | 0.692 | 0.697 |
| 8 | 0.760 | 0.762 | 0.763 | 0.764 | 0.760 | 0.762 |
| 9 | 0.931 | 0.917 | 0.931 | 0.919 | 0.931 | 0.918 |
| 10 | 0.906 | 0.908 | 0.912 | 0.911 | 0.906 | 0.908 |
| 11 | – | 0.930 | – | 0.932 | - | 0.930 |
| 12 | – | 0.938 | – | 0.939 | - | 0.938 |
| All loadings were significant considering p < 0.01 | | | | | | |
